# Judgments of predictability, preventability and causation of mental health homicides in England 2010 – 2023

**DOI:** 10.1177/00258024251363094

**Published:** 2025-08-03

**Authors:** Mayura Deshpande, Julia MA Sinclair, David S Baldwin

**Affiliations:** 1 Faculty of Medicine, 7423 University of Southampton, Southampton, UK

**Keywords:** Predictability, preventability, causation, mental health homicides, independent inquiry, mental health policy

## Abstract

Independent mental health homicide inquiries in England are required to comment on predictability and preventability and attribute causation. National commissioning bodies do not provide definitions. This study examines how predictability and preventability were determined, and causality attributed, by independent mental health homicide inquiries published in England between 2010 and 2023. The conceptual underpinnings of predictability and preventability in other specialities of medicine, and in suicide and homicide assessments in psychiatry are examined. We found 189 independent reports relating to mental health homicides, of which 162 independent homicide inquiries were included in the final analysis. No inquiry described how it attributed causation or addressed cognitive biases. A total of 130 of the 162 inquiries (80%) commented on either predictability or preventability: of these, only eight (6%) included a clear definition of predictability and preventability. Homicides were deemed predictable if the inquiry panel felt that the perpetrator's words or actions should have alerted professionals to a risk of significant violence; and preventable if the clinical team had knowledge, legal means, and opportunity to stop the homicide from occurring. 105 inquiries (81%) provided a firm view on both predictability and preventability. Of these, four homicides (4%) were deemed to be both predictable and preventable, ten (9%) were preventable but not predictable, five (5%) were predictable but not preventable, and 86 (82%) were neither predictable nor preventable. The implications of these findings are discussed, with recommendations to national commissioning bodies.

## Introduction

Homicides perpetrated by patients in receipt of care from mental health services in England are required to be subject to independent inquiry. Such inquiries are widely influential, with impact on public policy, clinical practice and the perception of safety and quality in mental health services.^1–3^ However, their effectiveness has been debated, and questions asked regarding both their methodology – which relies on the approach of root cause analysis – and their findings.^4–6^

Mental health homicide inquiries in England are expected to comment specifically on the predictability and preventability of the homicide, to make causal attributions, and to make recommendations to reduce recurrence of similar events in the future.^7^

This study aims to examine findings relating to predictability, preventability and causal attributions from mental health homicide inquiries in England over a 13-year period.

### Mental health homicides

These are homicides committed by individuals who were in receipt of treatment and care from mental health services, either at the time of the homicide or in the preceding period. The Office of National Statistics (ONS) lists the numbers of homicides in the general population each year,^8^ of which 11% are estimated to have been committed by individuals with a mental disorder for the period 2012 to 2022.^9^ The number of homicides in England and Wales in the general population and the proportion of these due to mental disorder was strongly correlated between 1957 and 1980, with both rising until they peaked in 1973; however, homicides due to mental disorder declined from 1981 to 2004, with a negative correlation between these and homicides in the general population.^10^ The ONS 2024 dataset shows that homicide rate remains low, with 9.5 homicides recorded per million population during the year ending March 2024; of these, around 5% of the perpetrators each year are convicted of manslaughter on the basis of diminished responsibility due to mental disorder.^8–10^

### Mental health homicide investigations

The history of English mental health homicides has been described comprehensively.^1^ A series of public inquiries led the Department of Health (now the Department of Health and Social Care, DHSC) to a policy position that all homicides by psychiatric patients would be investigated by an independent inquiry. This was formalised in 1994 in a circular entitled ‘Guidance on Discharge of Mentally Disordered People and Their Continuing Care in the Community Health Service Guidance 94 (27)’ (HSG (94) 27), which required independent inquiries to examine the patient's care, its suitability, standards, adequacy and the exercise of professional judgement.^11^ This was updated in 2005^12^ and again in 2015 in the Serious Incident Framework,^13^ Appendix 1 of which described the approach to investigating homicides by patients in mental health services. Among other things, it requires investigations to ‘…facilitate further examination of the care and treatment of patients in the wider context and establish whether or not an incident could have been predicted, or prevented, and if any lessons can be learned for the future to reduce the chances of recurrence…’

The 2015 Serious Incident Framework does not, however, provide definitions of predictability and preventability. NHS England, which assumed responsibility in 2013 for the commissioning of these investigations, has published other guidance documents periodically, but none have included definitions of preventability or predictability.^14,15^

### Predictability and preventability

The concepts of predictability and preventability are well-established in safety critical industries such as aviation^16^ and in some branches of medicine. The notion of prevention is of course central to much of public health in medicine, and to the criminal justice system, especially policing. However, no guidance is provided by NHS England on how to determine predictability and preventability of mental health homicides.

### Preventability and predictability in the criminal justice system

In the criminal justice system, it is stated that much policing resource is dedicated to preventing homicide but where previously the focus of prevention was on the more proximate causes of homicide (described as ‘being in the right place at the right time to intervene before a fatal blow is struck’), the focus in recent years has moved to examining a broader set of activities.^17^

A domestic violence related homicide (‘domestic homicide’) is defined as the death of a person aged 16 or over, resulting from violence, abuse or neglect by a person to whom he^1^ was related or with whom he was or had been in an intimate personal relationship, or a member of the same household as himself.^18^ Domestic homicides are subject to a statutory review process called a Domestic Homicide Review (DHR), which is a multi-agency review. DHRs are also required to comment on the extent to which the homicide could have been accurately predicted and prevented: however, no definitions are provided, nor guidance on how predictability and preventability may be ascertained.

### Preventability and predictability in general medicine

Where predictability and preventability appear to have widest application in medicine is in the field of adverse drug reactions (ADR). Literature in this field indicates a view that if an ADR is predictable, then it is preventable.^19,20^ ADRs that are extensions of known pharmacological effects and are dose-dependent are deemed to be more predictable than those that are idiosyncratic or due to immunological reactions.^21^

A number of criteria have been proposed to ascertain preventability, including when a drug is prescribed despite being known to be contra-indicated, when an inappropriate dose is prescribed, when there is lack of appropriate monitoring, when it is prescribed despite knowledge of interactions with other drugs, and when toxic serum concentrations are ignored.^22^

This has led to the development of two general methods of ascertaining predictability and preventability of ADRs – the judgement of one or more investigators, or the use of pre-defined explicit criteria – but neither is felt to be satisfactory.^23^ In this review of predictability and preventability of ADRs, the authors list several problems, including the weakness of consensus as a method (since experts can agree and yet be wrong), inadequacy of definition of standards of care, and circularity in several definitions of preventability. They note that attempts to list all preventable effects are bound to be incomplete and will not always apply to an individual case.

What is clear is that all notions and methods of predictability and preventability in ADRs hinge on a definite action that could or should have been taken, in which the prescriber or regulator has confidence that it would have stopped the ADR from occurring.^24^

Predictability and preventability are also explored in particular surgical specialities. For example, in a study examining the predictability and preventability of surgical readmissions, there was poor consensus between patients and doctors regarding predictability, preventability and risk factors and root causes of readmissions.^25^ Similarly, in another study examining predictability and preventability of shoulder dystocia during delivery of pregnancy, the authors concluded that the majority of shoulder dystocia cases occur without major risk factors; even the best antenatal predictors have a low positive predictive value, and shoulder dystocia therefore cannot be reliably predicted, with Caesarean delivery the only preventative measure, although the cause of other risks.^26^

### Preventability and predictability in suicide

Prediction and prevention, as noted previously, have received much attention in psychiatry, especially with the most serious adverse events such as suicide.^27^ The concept of prediction has a long and contentious history in psychiatry, which as a specialty is perhaps now in the ‘post-prediction’ world when it comes to suicide: the focus in mental health public policy is not on being able to predict suicide through the use of tools or validated instruments (which have low predictive validity), but on safety planning.^28^ Suicide prevention is seen as a public health imperative with a focus on community and population-level initiatives.^29^

A number of studies have examined the ability of psychiatrists to predict suicide,^30,31^ concluding that there is little evidence that death by suicide can be reliably predicted or prevented in clinical settings; that most suicide prediction models have low positive predictive values; and that while medication or psychotherapy can reduce suicide attempts, they do not reduce fatality,^32^ with the probable exception of clozapine and lithium.^33,34^

A possible explanation for the failure of instruments to predict suicide reliably is that suicide should be understood as a multifaceted phenomenon, which is the result of complex dynamic processes spanning psychological, sociological and biological dimensions.^35^

A study of a 15-year audit with semi-structured interviews shows that while a range of psychopathologies and suicidal cognitions were observed by clinicians at their last clinical contact with the patient, the immediate suicide risk was considered low or absent in the majority of cases.^36^ Therefore, use of instruments to predict suicide risk is being replaced by therapeutic risk assessment, formulation and risk management, including collaborative safety planning.^37^

What is striking is that, following decades of research, there is general consensus that suicide at an individual level is not amenable to prediction using the means and measures at our disposal, but there is value in preventative measures, aimed at the community or the population, the most effective of which is restricting access to means of harm.^6,29,38,39^

### Preventability and predictability in mental health homicides

A number of authors have examined the concepts of predictability and preventability of mental health homicides. A review in 1997 noted that we cannot predict risks accurately in clinical practice.^2^ Another review noted that, as with suicide, there is a trade-off between sensitivity and specificity of tools used to predict rare events like homicide.^5^ A review of the findings of the National Confidential Inquiry into Suicide and Homicide noted that even when assured of the confidentiality of the data they were providing, clinicians considered that 11 of the 15 inpatient homicides had not been preventable.^3^ This was reinforced by another review which found poor predictive validity of violence risk assessment tools used in the criminal justice system for sentencing.^40^

Therefore, there is much agreement that whilst we can identify potentially treatable factors that are associated with serious violence in schizophrenia and other psychoses, their clinical utility in violence risk assessment remains uncertain, and that tools purporting to predict the risk of severe violence such as homicide are unhelpful.^41^

In clinical practice, the focus is on identifying risk factors for violence in mental disorder, such as comorbid substance use, and attempt to modify these; other risk factors such as past history of criminality are obviously not amenable to modification.

Overall, therefore, while some other specialties within medicine emphasise the notions of predictability and preventability, there is little evidence in psychiatry that the most serious adverse events – suicide and homicide – can be accurately predicted at the individual level, and preventative efforts appear most effective when aimed at the population level, and specifically in suicide prevention, when they reduce access to means of self-harm.

We therefore aimed to understand how predictability and preventability were determined by independent mental health homicide inquiries in England.

## Method

We carried out a thematic analysis of all independent mental health inquiries relating to homicides in England between 2010 and 2023 published by NHSE on its website.^7^

This study was approved by the University of Southampton Research Ethics Committee, ERGO number 100030. Only material available to the public on the NHSE website and already in the public domain was included. Therefore formal NHS ethics committee approval was not required.

NHSE defines ‘mental health homicide’ as an act of homicide committed by a person in receipt of mental health services by an NHS-funded provider in England. It requires the investigation of such incidents to be undertaken by an organisation independent of the provider NHS organisation.^13,42^

Therefore, we included inquiry reports if they were of a completed homicide (either full reports or executive summaries) and were conducted by a body independent of the provider NHS organisation.

We identified 189 independent inquiry reports relating to mental health homicides on the NHSE website published between 2010 and 2023. Of these, one duplicate (published in two places) was excluded, leaving 188 reports: a further 26 were excluded, as they either did not relate to mental health homicides, or were related to a homicide but represented quality assurance, or desk-top reviews, as these are not investigations. A total of 162 homicide inquiry reports were included in the final document analysis.

Document analysis is a systematic procedure for reviewing or evaluating documents, used to provide context, generate questions, and track change over time; it can include both quantitative and qualitative components. We used a specific document analysis technique (‘READ’) recommended in health policy research.^43^ This includes discrete steps of making the material ready, extracting data, analysing data and distilling findings.

After analysing approximately 50% of the sample, to examine the level of agreement on all the characteristics we thematically analysed, a review of test–retest reliability was completed based on a sample of 15% of inquiry reports. There was 98% agreement. After the entire sample of 162 reports was analysed, an inter-rater reliability exercise was undertaken based on a random sample of 15% of inquiry reports: this found 92.9% agreement.

For the purposes of this study, we examined inquiry reports’ conclusions regarding predictability and preventability, definitions used and acknowledgement of challenges and biases.

## Results

The 162 Mental Health Homicide Inquiries (MHHI) described the deaths of 168 victims by 162 individual perpetrators in receipt of clinical care by NHS-funded mental health services. There were 156 single homicides and 6 double homicides.

Characteristics of perpetrators, victims, the homicide and the individual homicide inquiry reports from this study are described elsewhere.^44^ In this study, we focus on an examination of inquiry reports’ findings regarding predictability and preventability.

As noted earlier, NHSE and DHSC provide no definition of these terms, or guidance as to how predictability and preventability should be determined by independent mental health homicide inquiry panels.

A total of 130 of the 162 inquiries (80%) commented on either predictability or preventability, or both: 32 inquiries (20%) did not mention predictability or preventability. Of the 130 that did comment on either predictability or preventability, only 8 (6%) included a clear definition of predictability as well as preventability. None included a definition of either only preventability or only predictability.

### Definitions of predictability

See Table 1 for definitions of predictability and preventability used by inquiry reports.

**Table 1.** Definitions of predictability and preventability.

No	Predictability	Preventability
1	The quality of being thought likely to happen; predictable means that the probability was high enough to expect intervention or action by professionals to attempt to stop it	Prevention – to stop or hinder something from happening especially by advance planning or action… Preventability – there would have been knowledge, legal means and opportunity to stop it from happening…
2	The quality of being regarded as likely to happen, as a behaviour or an event. An essential characteristic of risk assessment is that they involve estimating a probability. If a homicide is judged to have been predictable, it means that the probability of violence, at the time, was high enough to warrant action by professionals to try to avert it	Prevention – to stop or hinder something from happening especially by advance planning or action and implies anticipatory counteraction; therefore for a homicide to have been preventable, there would have been knowledge, legal means and opportunity to stop it from happening…
3	If there was evidence from the perpetrator's words, actions or behaviour that should have alerted professionals that there was a real risk of significant violence, even if this evidence had been un-noticed or misunderstood at the time it occurred	If there were actions that the healthcare professionals should have taken, which they did not take, that could in all probability have made a difference to the outcome. Simply establishing that there were actions that could have been taken or opportunities that were missed would not provide evidence of preventability, as there are always things that could have been done better
4	If there was evidence from the perpetrator's words, actions or behaviour that should have alerted professionals that there was a real risk of significant violence, even if this evidence had been un-noticed or misunderstood at the time it occurred	If professionals had the knowledge, the legal means and the opportunity to stop the violent incident from occurring but did not take steps to do so. Simply establishing that there were actions that could have been taken would not provide evidence of preventability, as there are always things that could have been done to prevent any tragedy
5	The quality of being regarded as likely to happen, as a behaviour or an event. An essential characteristic of risk assessment is that they involve estimating a probability. If a homicide is judged to have been predictable, it means that the probability of violence, at the time, was high enough to warrant action by professionals to try to avert it	Prevention – to stop or hinder something from happening especially by advance planning or action and implies anticipatory counteraction; therefore for a homicide to have been preventable, there would have been knowledge, legal means and opportunity to stop it from happening…
6	If there was evidence from the service user's words, actions or behaviour that should have alerted professionals that there was a real risk of significant violence, even if this evidence had been un-noticed or misunderstood at the time it occurred	If there were actions that the healthcare professionals should have taken, which they did not take, that could in all probability have made a difference to the outcome. Simply establishing that there were actions that could have been taken or opportunities that were missed would not provide evidence of preventability, as there are always things that could have been done better. This is a significant legal threshold
7	The quality of being regarded as likely to happen, as a behaviour or an event. An essential characteristic of risk assessment is that they involve estimating a probability. If a homicide is judged to have been predictable, it means that the probability of violence, at the time, was high enough to warrant action by professionals to try to avert it	Prevention – to stop or hinder something from happening especially by advance planning or action and implies anticipatory counteraction; therefore for a homicide to have been preventable, there would have been knowledge, legal means and opportunity to stop it from happening…
8	The quality of being regarded as likely to happen, as a behaviour or an event. An essential characteristic of risk assessment is that they involve estimating a probability. If a homicide is judged to have been predictable, it means that the probability of violence, at the time, was high enough to warrant action by professionals to try to avert it	Prevention – to stop or hinder something from happening especially by advance planning or action and implies anticipatory counteraction; therefore for a homicide to have been preventable, there would have been knowledge, legal means and opportunity to stop it occurring

Most definitions used were broadly similar, with predictability mostly being defined in terms of likelihood or probability of a future event. Five of the eight definitions stated that the homicide would have been predictable ‘…if the probability was high enough to expect intervention or action by professionals to attempt to stop it…’ This is a description of predictability in a paper on the role of risk assessments in reducing the risk of homicides by people with mental illness,^45^ which states that ‘…a characteristic of risk assessments is that they involve estimating a probability – a degree of risk…so, when in our analysis, a report is classified as having concluded that the homicide was predictable, it means that the inquiry panel thought that the probability of violence, at the time, was high enough to warrant action by professionals to try to avert it…’

Three of the eight inquiries which included definitions of predictability and preventability focused on whether the perpetrator's words, actions or behaviours should have alerted professionals that there was a real risk of significant violence, even if this evidence had been un-noticed or misunderstood at the time it occurred. It is unclear where this definition is derived from, as these inquiry reports contain no references.

### Definitions of preventability

All eight inquiry reports which contained a definition of preventability said that a homicide would have been preventable if the clinical team had knowledge, legal means and opportunity to stop the homicide from occurring.

One inquiry stated that the homicide would be preventable ‘…if there were actions that the healthcare professionals should have taken, which they did not take, that could in all probability have made a difference to the outcome. Simply establishing that there were actions that could have been taken or opportunities that were missed does not necessarily provide evidence of preventability, as there are always things that could have been done better. This is a significant legal threshold…’ However, it is unclear what legal threshold the report is referring to, as there is no reference provided. It is similarly unclear what threshold of probability the inquiry panels had in mind.

### Findings regarding predictability and preventability

Of the 130 reports commenting on predictability and/or preventability, 17 homicides (13%) were deemed by the inquiry panel to be predictable (comprising 11 that were ‘definitely’ predictable and 6 that were ‘maybe’ predictable). Thirty-seven (28%) were deemed to be preventable (comprising 17 that were ‘definitely’ preventable and 20 which were ‘maybe’ preventable). Ten of the 17 deemed predictable were also thought to be preventable, and 10 of the 37 thought to be preventable were also considered predictable. When seen in the context of the entire sample of 162, the proportion of homicides that were deemed predictable falls to 10% and those deemed preventable falls to 23%.

As seen in Table 2, altogether, 105 (81%) provided a firm view on both predictability and preventability. Of these, 4 homicides (4%) were deemed to be both predictable and preventable, 10 (9%) were preventable but not predictable, 5 (5%) were predictable but not preventable, and 86 (82%) were neither predictable nor preventable.

**Table 2.** Preventability and preventability of homicides.

N = 105		Predictable	
		Yes	No
Preventable	Yes	4 (4%)	10 (9%)
	No	5 (5%)	86 (82%)

These findings contrast with those seen of an analysis in 2000 which found that of 40 inquiry reports between 1988 and 1997, 11 (27.5%) had been deemed predictable and 26 (65%) were deemed preventable.^45^

### Determination of predictability and preventability by inquiries

In the present sample, several inquiry reports attempt to describe their analysis. For instance, one inquiry report states that ‘…the incident was not predictable and therefore not preventable…’ Other inquiries move away from this, treating predictability and preventability as separate rather than conditional constructs, so that of the 113 reports that were deemed ‘definitely not’ predictable, 10 were deemed preventable. Most of these inquiries stated that the homicide would have been prevented had the perpetrator been treated more assertively – the types of treatments and interventions mentioned included the use of the antipsychotic clozapine, the use of the Community Treatment Order to enforce compliance with medication and follow-up and admittance to a psychiatric hospital.^46^ It is striking however that all refer to the preventability of serious violence by the perpetrator, rather than the act of homicide.

The same trend was seen in the 86 inquiry reports that deemed that the homicide was neither predictable nor preventable, with panels making statements to the effect that further violence by the perpetrator could have been predicted, or that the general risk of future violence was predictable, whereas the homicide itself could not have been foreseen.

Some inquiries appeared to consider the legal outcome of the trial as an indicator of preventability of the homicide; one inquiry report states that as the perpetrator did not succeed in mounting a defence of diminished responsibility^47^ at trial, this proved that he would not have met criteria for detention under the Mental Health Act 1983 prior to the homicide, and therefore the homicide was not preventable. Another noted that as the coroner's inquest, which took place before the inquiry report was finalised, had deemed the homicide predictable and avoidable, so too had the independent inquiry.

## Discussion

This study examines notions of predictability and preventability of homicides by people with mental disorders who are in care of mental health services, as described in independent inquiry reports. In our sample of 162 independent mental health homicide inquiries published in England between 2010 and 2023, only 10% were deemed predictable and 23% preventable; this is with the inclusion of ‘maybe’ predictable and ‘maybe’ preventable cases. When only those inquiries that had a definite view on both predictability and preventability are considered, only 4 homicides (4%) were deemed both predictable and preventable.

Although 130 reports commented on predictability and/or preventability, only 8 (6%) had definitions. In the absence of any nationally described definitions or criteria for determining predictability or preventability, these inquiry panels employed variable definitions, which, on further analysis, suffer from a number of shortcomings.

When inquiries defined predictability as an estimate of probability or likelihood, they did not address the issue of how the probability or likelihood of the perpetrator committing a homicide should be estimated. They did not specify at what threshold of probability or likelihood professionals should have taken action, or how this should be determined.

All inquiries examined the probability of serious violence rather than homicide, presumably because the literature on risk factors in mental disorder focuses on the risk of violence, rather than specifically homicide, such as previous history of violence, substance use or alcohol use.^48,49^ These risk factors are of course not specific to homicide; although studies find that most perpetrators of homicide in the general population and amongst those with mental disorder had a previous history of violence,^50^ many more people with histories of interpersonal violence do not go on to commit homicide, even with untreated mental illness. Violence risk assessment tools based on these risk factors generally have low predictive values in general psychiatric settings.^51,52^ Therefore, inquiries appeared to extrapolate an association between certain risk factors and violence in people with mental disorder to a completed act of homicide.

When inquiries defined the notion of preventability, they tended to stipulate that professionals would have had to have the knowledge, legal means and the opportunity to stop the homicide from occurring. The idea of ‘knowledge’ here appears to be that professionals would have to know what the perpetrator was thinking, or have explicit knowledge of his actions prior to the homicide, or explicit knowledge of his intention, which would alert professionals to an imminent risk. This suggests that many inquiries saw preventability as conditional, at least to some extent, on predictability.

In determining preventability, inquiries focused on events most proximate to the final act of homicide, and appeared to examine whether at that point, professionals might have done something that might have changed the course of events, which has been described as the tendency to consider events that occur later in a temporal chain as more mutable, or changeable, than those that occur earlier in the same sequence, even when both events are equally capable of changing the outcome.^53^ However, no inquiries explicitly acknowledged the role of counterfactuals, despite generation of counterfactual data being key to constructing an alternative version of events.^54^ Instead, they constructed a retrospective narrative of events, with a linear chain of events, where one event led in a straightforward fashion to another; identified omissions or sometimes commissions; picked one or more omission or commissions which they deemed to be significant or vital in the causal chain; and from this, arrived at views regarding predictability and preventability. This is a significant weakness of inquiries, as such narratives of omissions and commission can immediately attract counterfactual narratives.^55^

The underlying assumption appears to be that causes lead to effects in a simple, linear manner; this is highly unlikely to be the case in mental health homicides. Literature on accidents and safety science in industry and in health in the past 30 years shows that there is no universal model that explains all accidents or adverse events,^56^ and that healthcare, as a complex adaptive system, should be understood in terms of nonlinear, emergent dynamics; that unpredictability and paradox are pervasive; and that some things remain unknowable.^57^

In addition, it remains unclear what inquiries thought of the inter-relationship between predictability and preventability. Seventeen homicides in this sample were deemed predictable, of which 10 were also preventable. The remaining 7 of the 17 inquiry reports do not explain why they thought the homicide, despite being predictable, was not preventable. Similarly, inquiry reports do not explain why they consider some homicides to be preventable but not predictable. An event may be predictable (although there is little evidence that an individual homicide can be predicted) and not preventable. As described earlier, the literature on suicide indicates that there is more value in prevention through population measures than prediction at an individual level. There is no requirement for mental health inquiries to use legal thresholds of burden of proof.^58^ However, these legal thresholds are useful in attempting to understand the decision making of mental health homicide inquiries. None of the inquiries made clear whether they applied a specific legal burden of proof.^58^ Even the four inquiries which deemed the homicide to have been both predictable and preventable did not specify whether this was on the balance of probability (the so-called civil burden of proof) or beyond reasonable doubt (the so-called criminal burden of proof) – instead, they all listed multiple complex factors that were operating at the time of the homicide.

These inquiries are not statutory, nor are they trials in a court of law; however they operate as quasi-statutory processes, and professionals whose practice is criticised have neither right of reply nor appeal. Furthermore, families of the victim similarly may disagree with the conclusion of the inquiry but their views are seldom captured.^59^

Mental health homicide inquiries may benefit from clarity regarding their relationship with statutory processes such as the coroner's inquest. Inquests are legal inquiries into the cause and circumstances of a death, and are limited, fact-finding inquiries which usually take place after the criminal trial has concluded, and will reach a verdict of unlawful killing.^60^ A Prevention of Future Deaths Order can be issued if the inquest finds failings in treatment and care.^61^ Can an independent mental health homicide inquiry decide that the homicide was predictable and/or preventable if the inquest found no such failings?

Some inquiries did acknowledge the risk of cognitive biases, especially *hindsight bias*^62^ – the tendency to erroneously perceive events as inevitable or more likely once they have occurred – and added a statement that efforts had been made to reduce this bias, although they did not explain how they did so. However, no inquiries addressed other possible biases or important sources of bias such as terms of reference. Other important biases to consider might include:

1. *Anchoring bias*^63,64^ – the biasing of decisions towards previously acquired information, which in homicide inquiries might result in panel members being unduly influenced by findings of previous mental health homicide inquiry reports, the ‘What-You-Look-For-Is-What-You-Find’ principle.
2. *Framing bias*^65^ – the tendency to base decisions on the way the information is presented (with attendant positive or negative connotations), as opposed to just on the facts themselves, which in homicide inquiries might result in panel members being unduly influenced by the findings of the provider's internal investigation report, or even the terms of reference.
3. *Confirmation bias*^66^ – the tendency to select, interpret, focus on and remember information in a way that confirms one's preconceptions, views, and expectations.
4. *Outcome bias*^62^ – the tendency to judge the quality of thinking and the competence of the decision-maker more highly when the decision is followed by success than when it is followed by a failure. This was not mentioned in any inquiry, although this bias and hindsight bias may be presented as counterfactual narratives to nearly all conclusions of any independent mental health homicide inquiry.

Perhaps the most striking feature is that no inquiry challenged the presumption that predictability and preventability can be determined retrospectively, despite overwhelming consensus in literature that tools to predict homicide have poor positive predictive value.^2,5,40^ Previous reviews of homicide inquiries have found that these individual inquiries cannot show that specific service failures cause homicides^1,2,5,67^; that a degree of retrospective causal indeterminacy always applies as links between retrospective events are usually indeterminate, with the risk of creeping determinacy^5^; that at best, these inquiries make subjective judgements about whether any aspects of clinical care or service provision contribute to homicides,^1,3,6,55^ but may be interpreted as being causative; and that inquiry panels themselves have acknowledged that even best practice is no guarantee of prevention.^68^

## Conclusion

Independent mental health homicide inquiries have a major influence on mental health policy and the public perception of risk, and statements regarding predictability and preventability, more so than any other parts of inquiry reports, are often quoted in the media. As this study shows, the notion of determining preventability and predictability of a mental health homicide retrospectively through an inquiry process is beset with conceptual and methodological weaknesses. It is unclear therefore what value these notions add to mental health homicide inquiries.

We recommend that if these inquiries are to continue, that the requirement for panels to comment on predictability and preventability is removed. Alternatively, if this requirement is to persist, then we recommend that explicit definition and thresholds are provided by NHS England. In addition, mental health homicide inquiry panels should consider whether they feel able to comment on predictability and preventability, and to be more transparent about their decision making.

## Notes

1. Although the masculine pronoun is used in the Domestic Violence, Crime and Victims Act 2004, 65.4% of the victims of domestic homicide were female for year ending March 2023 (Office of National Statistics, 2024).
